# Targeting cariogenic pathogens and promoting competitiveness of commensal bacteria with a novel pH-responsive antimicrobial peptide

**DOI:** 10.1080/20002297.2022.2159375

**Published:** 2022-12-20

**Authors:** Wentao Jiang, Zhuo Xie, Shuheng Huang, Qiting Huang, Lingling Chen, Xianling Gao, Zhengmei Lin

**Affiliations:** Hospital of Stomatology, Guangdong Provincial Key Laboratory of Stomatology, Guanghua School of Stomatology, Sun Yat-sen University, Guangzhou, Guangdong China

**Keywords:** Antimicrobial peptide, dental caries, Ph-response, biofilm, *streptococcus mutans*, *streptococcus gordonii*

## Abstract

Novel ecological antimicrobial approaches to dental caries focus on inhibiting cariogenic pathogens while enhancing the growth of health-associated commensal communities or suppressing cariogenic virulence without affecting the diversity of oral microbiota, which emphasize the crucial role of establishing a healthy microbiome in caries prevention. Considering that the acidified cariogenic microenvironment leads to the dysbiosis of microecology and demineralization of enamel, exploiting the acidic pH as a bioresponsive trigger to help materials and medications target cariogenic pathogens is a promising strategy to develop novel anticaries approaches. In this study, a pH-responsive antimicrobial peptide, LH12, was designed utilizing the pH-sensitivity of histidine, which showed higher cationicity and stronger interactions with bacterial cytomembranes at acidic pH. *Streptococcus mutans* was used as the *in vitro* caries model to evaluate the inhibitory effects of LH12 on the cariogenic properties, such as biofilm formation, biofilm morphology, acidurance, acidogenicity, and exopolysaccharides synthesis. The dual-species model of *Streptococcus mutans* and *Streptococcus gordonii* was established *in vitro* to evaluate the regulation effects of LH12 on the mixed species microbial community containing both cariogenic bacteria and commensal bacteria. LH12 suppressed the cariogenic properties and regulated the bacterial composition to a healthier condition through a dual-functional mechanism. Firstly, LH12-targeted cariogenic pathogens in response to the acidified microenvironment and suppressed the cariogenic virulence by inhibiting the expression of multiple virulence genes and two-component signal transduction systems. Additionally, LH12 elevated H_2_O_2_ production of the commensal bacteria and subsequently improved the ecological competitiveness of the commensals. The dual-functional mechanism made LH12 a potential bioresponsive approach to caries management.

## Introduction

From the perspective of the ecological plaque hypothesis, the development of dental caries is a consequence of the dysbiosis of oral microbial communities, the acidification of the localized microenvironment, and the disequilibrium of enamel mineral homeostasis [1]. Cariogenic pathogens, such as *Streptococcus mutans*, utilize dietary carbohydrates to produce exopolysaccharides (EPS) and acidic by-products to promote the cariogenic properties of dental plaque biofilms [2]. On the contrary, commensal bacteria can counter the establishment of the cariogenic biofilms. For example, *Streptococcus gordonii* and *Streptococcus sanguinis* can antagonize the growth and virulence of cariogenic pathogens and promote the ecological advantages of the commensals by H_2_O_2_ production and alkali production, which are always associated with sound teeth surfaces and lower caries risk [3,4]. Conventional antimicrobial caries prevention strategies narrowly aim at killing oral bacteria and eliminating oral biofilms eradicate cariogenic bacteria and commensal bacteria indiscriminately [5]. Novel ecological approaches to caries prevention focus on targeting cariogenic bacteria without disturbing other resident microbiota and suppressing cariogenic virulence while maintaining the viability of commensal plaque microflora [5,6].

Antimicrobial peptides are promising anticaries agents with experimentally perceived effects on cariogenic bacteria and biofilms; however, the broad-spectrum activity may disrupt oral microecological balance, which suggests the importance of modifying antimicrobial peptides with the properties of precision targeting and microecology regulating for caries management [6,7]. The pathological microenvironments of tumor or infection sites are more acidic than healthy tissues, rendering them attractive triggers for bioresponse [8,9]. These biological signals promote the precision and efficacy of the bioresponsive materials and medications [9]. For example, a class of pH-responsive antimicrobial peptides were designed for *Helicobacter pylori* infection, which showed potent antibacterial activity against *H. pylori* in the acidic environment of stomach and specifically targeted the pathogens without harming the commensal bacteria [10]. Considering that the acidified microenvironment is a main contributor to the microecology dysbiosis and enamel demineralization in dental caries, the pH difference between healthy oral cavity and acidified cariogenic biofilms is a potential trigger for the bioresponsive anticaries agent.

In this study, a pH-responsive antimicrobial peptide, LH12 (Gly-Leu-Leu-His-Leu-Leu-His-His-Leu-Leu-His-His-NH_2_), was designed utilizing the pH-sensitivity of histidine to explore the effects and mechanisms of the pH-responsive antimicrobial peptide on targeting cariogenic pathogens and regulating cariogenic biofilms [11]. It was speculated that the histidine-rich sequence of LH12 could potentiate its antimicrobial activity at the cariogenic acidic pH and this pH-responsive property could help it target cariogenic bacteria in response to their acidified metabolic microenvironment. Our results will demonstrate the potential of the pH-responsive antimicrobial peptide as a novel ecological caries prevention approach and highlight the prospect of bioresponsive medications in controlling infectious diseases with multispecies microbiota and particular pathological microenvironments.

## Materials and methods

### Peptides and chemicals

LH12 was synthesized by Ontores Biotechnologies (China). Hydrophobic moment (μH), hydrophobic ratio and helical-wheel diagram were determined at Heliquest (https://heliquest.ipmc.cnrs.fr). Net charges at different pH values were calculated at NovoPro (https://novopro.cn/tools/calc_peptide_property.html). The peptide structure was predicted using I-Tasser server (https://zhanggroup.org/I-TASSER/) [12] and rebuilt with Swiss-PdbViewer 4.1.0.

The primers and fluorescent probes were synthesized by TsingKe BioTech (China). Unless otherwise stated, chemicals were purchased from Macklin Biochemical (China).

### Bacterial inoculation and biofilm cultivation

Bacterial strains were obtained from Guangdong Province Key Laboratory of Stomatology. *Streptococcus mutans* UA159, *Streptococcus sanguinis* JCM 5708, *Streptococcus gordonii* DL1, *Streptococcus mitis* ATCC 6249, *Streptococcus salivarius* ATCC 27,945, *Actinomyces viscosus* ATCC 15,987, *Actinomyces naeslundii* JCM 8349, *Lactobacillus casei* ATCC 393, *and Lactobacillus fermentum* A1753 were grown in brain heart infusion (BHI, Tuopu, China) broth anaerobically at 37°C [13]. *Escherichia coli* K12 and *Staphylococcus aureus* ATCC 6538 were grown in Luria-Bertani broth (Tuopu, China) aerobically at 37°C [14].

Saliva collected from three volunteers with ethical approval of Guanghua School of Stomatology, Sun Yat-sen University (KQEC-2021-033) was pooled and sterilized by filters (0.22 μm; Millipore, USA). Saliva (50 μL) was added in 24-well plates to allow saliva pellicle formation at 37°C for 2 h.

*Mutans (10^6^ CFU/mL) was used for single-specie biofilms, while S. mutans (10^6^ CFU/mL) and S. gordonii (10^6^ CFU/mL) were used for dual-species biofilms.*

① *S. mutans* were incubated in BHI broth containing 1% sucrose (BHIS) at 37°C for 16 h anaerobically. The 16-hour-old biofilms were used for Live/Dead staining.

② *S. mutans* were incubated in BHIS containing serially diluted LH12 at 37°C for 24 h anaerobically. The 24 h-old biofilms were used for biofilm formation assay.

**Figure 1.**
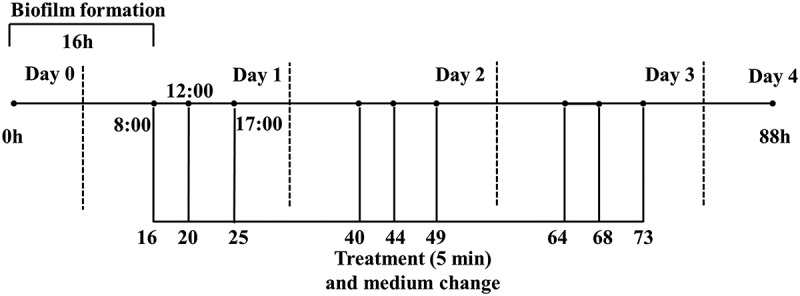
The short-term treatment on the preformed biofilms.

③ *S. mutans* were incubated in BHIS containing 1% sucrose at 37°C for 16 h anaerobically and the 16-hour-old biofilms were exposed to the short-term treatment three times daily (Figure 1). The preformed single-specie biofilms were rinsed and treated with serially diluted LH12 for 5 min at 16 h, 20 h, 25 h, 40 h, 44 h, 49 h, 64 h, 68 h, 73 h and 88 h. After each treatment, the biofilms were rinsed and fresh medium was added. After the final treatment, the biofilms were used for lactic acid measurement, water-insoluble EPS measurement, CFU counting and morphology observation.

④ *S. mutans* and *S. gordonii* were incubated in BHIS containing serially diluted LH12 at 37°C for 24 h anaerobically. The 24-hour-old biofilms were used for biofilm formation assay, biofilm strength measurement and CFU counting.

⑤ *S. mutans* and *S. gordonii* were incubated in BHIS containing 1% sucrose at 37°C for 16 h anaerobically and the 16-hour-old biofilms were exposed to the short-term treatment three times daily (Figure 1). The preformed dual-specie biofilms were rinsed and treated with serially diluted LH12 for 5 min at 16 h, 20 h, 25 h, 40 h, 44 h, 49 h, 64 h, 68 h, 73 h and 88 h. After each treatment, the biofilms were rinsed and fresh medium were added. After the final treatment, the biofilms were used for lactic acid measurement, water-insoluble EPS measurement, CFU counting and fluorescence in situ hybridization (FISH).

⑥ *S. mutans* and *S. gordonii* were incubated in BHIS at 37°C for 16 h anaerobically. The 16-hour-old biofilms were used for reactive oxygen species (ROS) detection.

### Bacterial susceptibility assay

The minimal inhibitory concentration (MIC) and minimal bactericidal concentration (MBC) were determined using a modified broth microdilution method [15]. The buffered BHI broth was prepared using 50 mM phosphate/citrate buffer (pH 5.5 and pH 7.2) and used for measuring antibacterial concentrations at different pH values [16]. The ordinary BHI broth was used for determining the MICs and MBCs of multiple caries-associated bacteria [17]. Broth or buffered broth, two-fold serial dilutions of LH12 and bacteria (10^6^ CFU/mL) were incubated in 96-well plated for 24 h. MIC was recorded as the lowest concentration where no visible growth of bacteria was inspected [18]. Aliquots (50 µL) taken from microwells were cultured on agar plates for 48 h. MBC was recorded as the lowest concentration with no colony formation [17]. The experiment was performed in triplicate and repeated six times.

### Biocompatibility assay

Human gingival epithelial cells (HGECs) were obtained from Guangdong Province Key Laboratory of Stomatology. HGECs were cultured in Dulbecco’s Modified Eagle’s Medium/Nutrient Mixture F-12 (Gibco, Australia) containing 10% fetal bovine serum (Gibco, Australia) and 1% penicillin – streptomycin solution at 37°C with 5% CO_2_ for 48 h. Cells were treated with or without LH12 for 45 min. Cells were rinsed with phosphate-buffered saline (PBS), 100 μL fresh medium and 10 μL CCK-8 reagent was added, and cells were incubated at 37°C for 2 h. Cellular morphology was observed using an inverted microscope (Zeiss, Germany). OD_450_ values were determined using a Synergy H1 multi-mode microplate reader (BioTek, USA). The experiment was performed in triplicate and repeated three times.

Hemolytic activity of LH12 was examined as described previously [17]. Sheep erythrocytes were collected by centrifugation (3000 rpm, 5 min) and were incubated in PBS containing LH12 at 37°C for 30 min. Triton X-100 (1%) was used as positive control and PBS was used as negative control. After centrifugation (3000 rpm, 10 min), OD_540_ values of the supernatants were recorded using a Synergy H1 multi-mode microplate reader. The experiment was performed in triplicate and repeated three times.

### Circular dichroism (CD) spectrum

LH12 was dissolved to 100 μg/mL in phosphate/citrate buffer or the buffer containing 50% trifluoroethanol (TFE). CD spectra were tested on a J-810 CD Spectrometer (JASCO, Japan). The measurement was performed in duplicate and repeated three times. Data were averaged over ten scans from 250 nm to 200 nm and expressed as the mean residue ellipticity *θ* (deg·mol-1·m-1) [17].

### Membrane potential measurement

Membrane depolarization activity was determined with 3,3’-dipropylthiadicarbocyanine iodide [DiSC3(5)] [19]. *S. mutans* (10^7^ CFU/mL) in phosphate/citrate buffer with or without LH12 were incubated with 4 μM DiSC3(5) for 30 min. Fluorescence intensity was recorded at excitation and emission wavelengths of 622 nm and 670 nm using a Synergy H1 multi-mode microplate reader. The experiment was performed in triplicate and repeated three times.

### Membrane permeabilization assay

Outer membrane permeabilization was measured using 1-N-phenyl-napthylamine (NPN) [20]. *E. coli* (OD_600_ = 0.1) in phosphate/citrate buffer with or without LH12 were mixed with 10 μM NPN. The increase of fluorescence intensity was monitored at excitation and emission wavelengths of 350 and 420 nm using a Synergy H1 multi-mode microplate reader. The experiment was performed in triplicate and repeated three times.

Inner membrane permeabilization was measured using *o*-Nitrophenyl-β-D-galactopyranoside (ONPG) [21]. *E. coli* was grown in Luria-Bertani broth with 2% lactose. Bacteria (10^8^ CFU/mL) in phosphate/citrate buffer with or without LH12 were mixed with 30 mM ONPG. The increase of OD_420_ values was monitored using a Synergy H1 multi-mode microplate reader. The experiment was performed in triplicate and repeated three times.

### Spot assay

Spot assay was conducted to evaluate the antibacterial effect at different pH values [22]. *S. mutans* (10^6^ CFU/mL) in buffered BHI broth with or without LH12 was incubated at 37°C anaerobically for 5 min, 15 min and 30 min. Aliquots (15 μL) were cultured on agar plates at 37°C anaerobically for 24 h. The experiment was performed in triplicate and repeated three times.

### Confocal laser scanning microscope (CLSM) observation

Biofilms were observed using an Olympus FV3000 confocal laser scanning microscope (Olympus, Japan) and displayed as maximum intensity projection of 3D z-stack image series. The experiments were performed in triplicate and repeated three times.

To evaluate the antibiofilm effect of LH12 at different pH values, the 16-hour-old *S. mutans* biofilms were treated in phosphate/citrate buffer with or without LH12 for 30 min and stained with a Live/Dead^TM^ BacLight^TM^ Bacterial Viability Kit (Invitrogen, USA) containing SYTO-9 and propidium iodide (PI). The laser power and gain were kept constant for images of the samples from the same batch. Biomass quantification was conducted on the open-source image analysis software ImageJ.

To observe the effect of LH12 on biofilm morphology and EPS accumulation, *S. mutans* biofilms exposed to the short-term treatment were stained with TRITC labeled concanavalin A (TRITC-ConA, Xarxbio, China) and SYTO-9 [23]. The thickness of biofilms was measured in random five sights.

To observe the bacterial composition of the dual-species biofilms exposed to the short-term treatment, FISH was conducted with specific probes as described previously [24]. The dual-species biofilms were fixed in 4% paraformaldehyde, rinsed with sterile water, and dried at 46°C. Samples were incubated in lysis buffer (50 mM EDTA, 100 mM Tris-HCl, pH 8.0) containing 30 mg/mL lysozyme at 37°C for 20 min. Biofilms were rinsed, serially dehydrated in 50%, 80%, and 100% ethanol for 3 min, dried at 46°C, and incubated in hybridization buffer (20 mM Tris-HCl, pH 8.0; 0.9 M NaCl; 20% formamide; 0.01% SDS) containing 2 nM-specific probes (Table 1) at 46°C for 90 min. Biofilms were rinsed with preheated wash buffer (20 mM Tris-HCl, pH 8.0; 5 mM EDTA; 215 mM NaCl; 0.01% SDS) at 48°C for 15 min and rinsed in nuclease-free water.

**Table 1.** Probes used in fluorescent in situ hybridization.

Probes	Sequences (5’-3’)
*S. mutans*	FAM-5’-ACTCCAGACTTTCCTGAC-3’
*S. gordonii*	Cy5-5’-ACTGTGCGTTCTACTTGC-3’

### Growth inhibition kinetics

Growth inhibition effects of LH12 were determined as described previously [24]. Bacteria (10^6^ CFU/mL) were inoculated in BHI broth with or without LH12 at 37°C anaerobically and OD_600_ values were monitored using a Synergy H1 multi-mode microplate reader. The experiment was performed in triplicate and repeated three times.

### Assays on cariogenic virulence

The effect of LH12 on biofilm formation was evaluated using crystal violet [25]. The 24-hour-old biofilms were fixed with methanol for 15 min and stained with 0.1% crystal violet for 5 min. The dye bound to the biofilms was resolubilized by ethanol. OD_595_ values were measured using a Synergy H1 multi-mode microplate reader. The experiment was performed in triplicate and repeated three times.

The biofilm strength of the dual-species biofilms was determined with crystal violet staining. The 24-hour-old dual-species biofilms were washed with PBS on a shaking table at 250 rpm for 3 min. The residual biofilms were stained as mentioned above. The experiment was performed in triplicate and repeated three times.

The acidurance was tested using CCK-8. Bacteria (10^8^ CFU/mL) were treated with or without LH12 for 5 min and resuspended in buffered BHI broth (pH 5.5, pH 5.0 and pH 4.5). Suspensions (100 μL) and CCK-8 reagent (10 μL) were incubated anaerobically at 37°C for 1 h. OD_450_ values were determined using a Synergy H1 multi-mode microplate reader. The experiment was performed in triplicate and repeated three times.

The lactic acid production of biofilms exposed to the short-term treatment was determined as described previously [26]. Biofilms were incubated in buffered peptone water (China) containing 0.2% sucrose at 37°C anaerobically for 2 h. Supernatants were tested with a lactate assay kit (Jiancheng, China). OD_570_ values were recorded using a Synergy H1 multi-mode microplate reader and lactate concentrations were calculated using a standard curve. The experiment was performed in triplicate and repeated three times.

The water-insoluble EPS production of biofilms exposed to the short-term treatment was determined as described previously [26]. Biofilms were thoroughly washed with sterile water. The precipitates were mixed with 0.4 M NaOH. After centrifugation (8000 rpm, 5 min), supernatants were mixed with anthrone-sulfuric acid reagent (95°C, 6 min). OD_625_ values were detected using a Synergy H1 multi-mode microplate reader and the concentrations of water-insoluble EPS were calculated using a standard curve. The experiment was performed in triplicate and repeated three times.

### CFU counting

CFU counts were calculated to evaluate the effects of LH12 on the viability of *S. mutans* in the single-specie biofilms exposed to the short-term treatment and on the bacterial composition of the dual-species biofilms. The experiments were performed in triplicate and repeated three times.

The single-specie biofilms exposed to the short-term treatment were dispersed in PBS by sonication and vortexing. Suspensions were cultured on BHI agar plates anaerobically at 37°C for 24 h.

The 24-hour-old dual-species biofilms and the dual-species exposed to the short-term treatment were dispersed in PBS by sonication and vortexing. Suspensions were cultured on BHI agar plates (broth for *S. mutans* and *S. gordonii*) and mitis-salivarius-bacitracin agar (Tuopu, China) plates (broth for *S. mutans* selectively) anaerobically at 37°C for 24 h.

### Quantitative real-time PCR

*S.mutans* and *S. gordonii* were cultured in BHI broth with or without LH12 for 30 min. RNA was isolated using an RNA-Quick Purification Kit (Yishan, China). Reverse transcription was performed using a PrimeScript^TM^ RT reagent Kit with gDNA Eraser (TaKaRa, Japan). Tested genes and specific primers are listed in Table 2 [24,26]. Quantitative real-time PCR was performed with Hieff® qPCR SYBR® Green Master Mix (Yeasen, China) on a LightCycler® 480-II system (Roche, Switzerland). The relative gene expression fold changes were calculated with 2^−ΔΔCt^ method. The experiment was performed in triplicate and repeated three times. More details were listed in Supplemental Material.

**Table 2.** Primers used in quantitative real-time PCR.

Primers		Sequences (5’-3’)
*S. m* 16S rRNA	F	AGCGTTGTCCGGATTTATTG
	R	CTACGCATTTCACCGCTACA
*ldh*	F	AAAAACCAGGCGAAACTCGC
	R	CTGAACGCGCATCAACATCA
*atpD*	F	TGTTGATGGTCTGGGTGAAA
	R	TTTGACGGTCTCCGATAACC
*gtfB*	F	CACTATCGGCGGTTACGAAT
	R	CAATTTGGAGCAAGTCAGCA
*gtfC*	F	GATGCTGCAAACTTCGAACA
	R	TATTGACGCTGCGTTTCTTG
*gtfD*	F	TTGACGGTGTTCGTGTTGAT
	R	AAAGCGATAGGCGCAGTTTA
*vicR*	F	CGTGTAAAAGCGCATCTTCG
	R	AATGTTCACGCGTCATCACC
*liaR*	F	CATGAAGATTTAACAGCGCG
	R	CGTCCTGTGGCACTAAATGA
*comD*	F	TTCCTGCAAACTCGATCATATAGG
	R	TGCCAGTTCTGACTTGTTTAGGC
*comE*	F	TTCCTCTGATTGACCATTCTTCTG
	R	GAGTTTATGCCCCTCACTTTTCAG
*S. g* 16S rRNA	F	AAGCAACGCGAAGAACCTTA
	R	GTCTCGCTAGAGTGCCCAAC
*spxB*	F	GGATGCTTTGGCTGAAGAC
	R	GGACCACCTGAACCTACTG

### Competition assay

Antagonism between *S. gordonii* and *S. mutans* was evaluated using competition assay [3]. Bacteria (10^7^ CFU/mL) were incubated in BHI broth with or without LH12 anaerobically at 37°C for 5 min. Aliquots (10 μL) of both strains were incubated beside each other on half-strength BHI agar plates anaerobically at 37°C for 24 h. The experiment was performed in triplicate and repeated three times.

### Measurement of H_2_O_2_ production

H_2_O_2_ production was determined with the indicator Prussian blue plates [3]. *S. gordonii* (OD_600_ = 0.1) was treated with or without LH12 for 5 min. Aliquots (5 μL) were cultured on plates anaerobically at 37°C for 24 h. The experiment was performed in triplicate and repeated three times.

### ROS detection

ROS generation was detected with 2′,7′-dichlorodihydrofluorescein diacetate (DCHF-DA) [27]. *S. gordonii (*10^8^ CFU/mL) and the 24-hour-old dual-species biofilms were incubated in BHI broth containing 10 μM DCFH-DA at 37°C anaerobically for 30 min and were treated with LH12 solution or sterile water for 5 min. Fluorescence intensity was monitored at excitation and emission wavelengths of 504 and 529 nm using a Synergy H1 multi-mode microplate reader. The experiment was performed in triplicate and repeated three times.

### Statistical analysis

Statistical analyses were performed with GraphPad Prism 6.0. Mann-Whitney test was used to determine if the MICs/MBCs at pH 5.5 were smaller than the values at pH 7.2. One-way ANOVA and Tukey’s multiple comparison test were used to determine the different effects of different peptide groups on cell viability, hemolysis, membrane depolarization activity, membrane permeabilization activity, biofilm formation, acidurance, lactic acid production, water-insoluble EPS synthesis, bacterial viability, biofilm thickness, biofilm strength, gene transcription, and ROS production. Two-way ANOVA and Tukey’s multiple comparison tests were used to compare the different effects of different peptide groups on the ratios of live bacteria and dead bacteria in Live/Dead staining images. Two-way ANOVA and Tukey’s multiple comparison test were also used to compare the different effects of different peptide groups on the CFU counts of *S. mutans* and *S. gordonii* in the dual-species biofilms. Differences were considered significant when *P* < 0.05.

## Results

### Characteristics and biocompatibility of LH12

**Figure 2.**
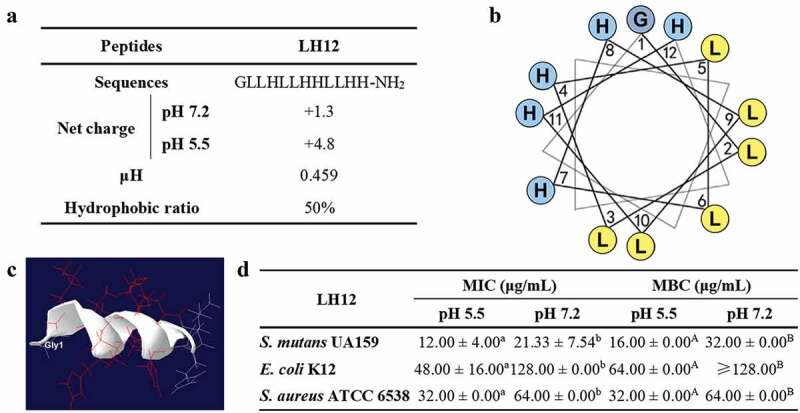
Characteristics of LH12. (a) amino acid sequence and physicochemical properties of LH12. (b) helical wheel diagram of LH12. Blue represents hydrophilic amino acid residues, whereas yellow represents hydrophobic amino acid residues. (c) molecular modelling of the structure of LH12. (d) antibacterial concentrations of LH12 at pH 5.5 and pH 7.2 on *S. mutans*, *E. coli* and *S. aureus* were tested to investigate the pH-responsive antimicrobial activity. Data are represented as mean ± standard deviation of six independent experiments. Different superscript lower case letters indicate significant differences between MICs at pH 5.5 and pH 7.2 (*P* <0.05). Different superscript capital letters indicate significant differences between MBCs at pH 5.5 and pH 7.2 (*P* <0.05).

Most natural antimicrobial peptides with good amphiphilicity possess a hydrophobic ratio from 40% to 60% and a μH from 0.3 to 0.6 [13]. As shown in Figure 2a, the μH value of LH12 was 0.459 and the hydrophobic ratio was 50%, which were within the desired range. The net charge of LH12 in pH 5.5 (the acidic pH where enamel demineralization occurs) was 4.8, while that in pH 7.2 (the neutral pH within the buffering range of saliva) was 1.3, suggesting that the acidic condition increased the cationicity of LH12. The helical wheel diagram (Figure 2b) shows that the hydrophobic sector was separate from the hydrophilic sector, indicating its potential amphiphilicity. The molecular modelling demonstrated the helical structure of LH12 visually (Figure 2c). To determine the pH-responsive antimicrobial activity of LH12, the antibacterial concentrations of LH12 at pH 5.5 and pH 7.2 were tested with the primary cariogenic pathogen (*S. mutans*) and two most common clinical pathogens (*S. aureus* and *E. coli*) (Figure 2d). LH12 displayed stronger antibacterial activity against *S. mutans*, *S. aureus* and *E. coli* at pH 5.5 with MICs and MBCs ranging from 12.00 μg/mL to 64.00 μg/mL, which were a half of the MICs and MBCs at pH 7.2 ranging from 21.33 μg/mL to more than 128.00 μg/mL. The acidic condition enhanced the antibacterial activity of LH12. Altogether, LH12 is an acid-activated pH-responsive cationic amphiphilic α-helical antimicrobial peptide.

**Figure 3.**
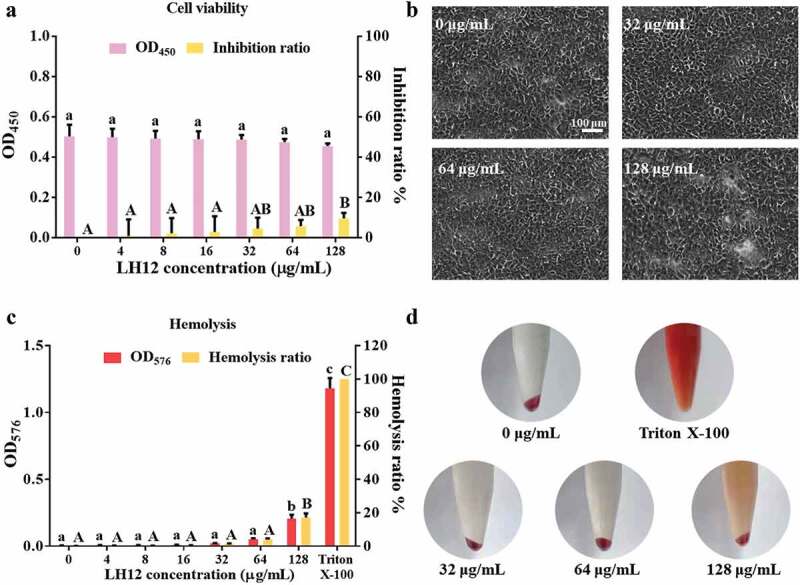
Biocompatibility of LH12. (a) cytotoxicity of LH12 on HGECs was tested with CCK-8 at OD_450_. Inhibition ratio = (OD_450-negative control_ − OD_450-tested well_)/OD_450-negative control_ ×100%. Data are represented as mean ± standard deviation. Different superscript letters indicate significant differences (*P* <0.05). (b) microscope images of the HGECs at the high concentrations of LH12. (c) hemolytic toxicity of LH12 was tested at OD_576_. Hemolysis ratio = (Od_576-tested well_ – OD_576-negative control_)/(OD_576-Triton X-100_ – OD_576-negative control_) × 100%. Data are represented as mean ± standard deviation. Different superscript letters indicate significant differences (*P* <0.05). (d) hemolysis images of negative control, positive control (Triton X-100) and groups of LH12 at high concentrations.

The pH-responsive property of LH12 may also alleviate safety concerns regarding its theoretical mild killing effects at physiological pH values [28]. To determine the compatibility of LH12, cytotoxicity assay on HGECs and hemolysis assay on mammalian erythrocytes were conducted. Only at a high concentration of 128 μg/mL would LH12 show slight cytotoxicity on HGECs with an inhibition ratio around 9.64% but without a significant reduction of the OD_450_ value after 45 min treatment (Figure 3a). Figure 3b shows the cellular morphology visually, in which no obvious cell destruction and no loss of adhesion were observed even at the high concentrations of LH12. As shown in Figure 3c, LH12 showed no hemolytic toxicity below 64 μg/mL and exhibited an increase of hemolysis ratio to 17.15% at 128 μg/mL. Figure 3d also demonstrates that LH12 did not significantly rupture erythrocytes at no more than 64 μg/mL and only showed visible hemolytic toxicity at 128 μg/mL. The results of cytotoxicity assay and hemolysis assay demonstrated the acceptable biocompatibility of LH12.

### Selective inhibition of LH12 on cariogenic bacteria

**Figure 4.**
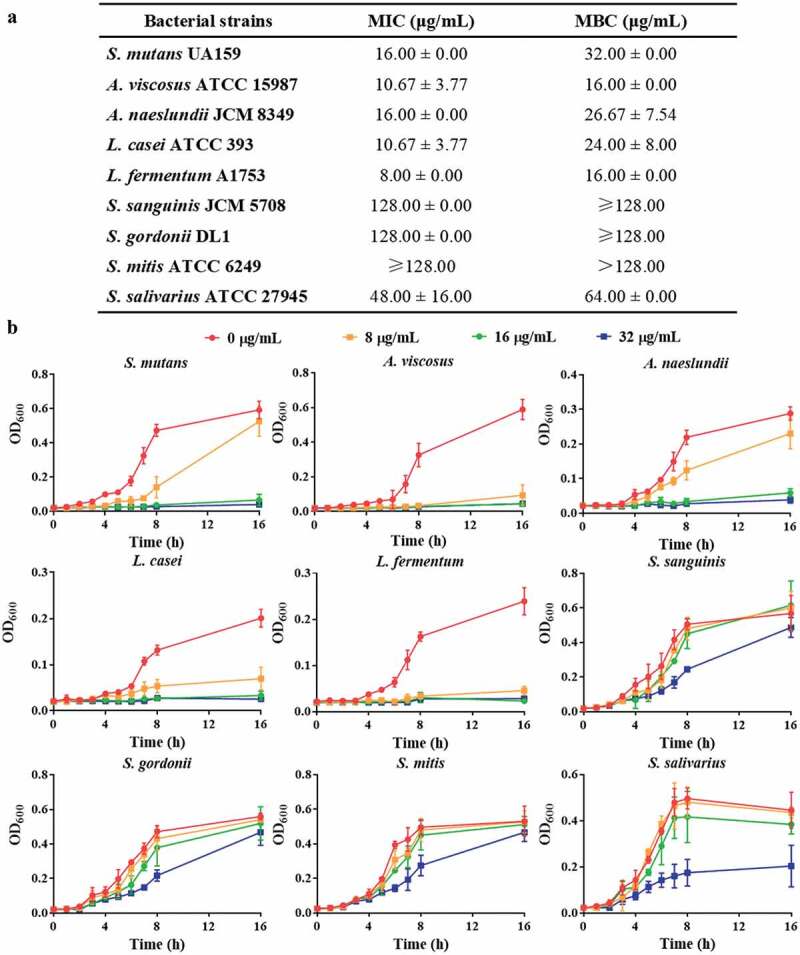
Selective inhibition of LH12. (a) MICs and MBCs of LH12 against the cariogenic bacteria and commensal bacteria. Data are represented as mean ± standard deviation of six independent experiments. (b) growth inhibition kinetics of LH12 on the cariogenic bacteria and commensal bacteria.

To verify the speculation that the pH-responsive antimicrobial peptide LH12 would be activated in the acidified microenvironment created by the cariogenic bacteria and subsequently targeted the pathogens without disturbing the commensals, the bacterial susceptibility assays and growth inhibition kinetics of multiple cariogenic bacteria (*S. mutans*, *A. viscosus*, *A. naeslundii*, *L. casei*, and *L. fermentum*) and commensal bacteria (*S. sanguinis*, *S. gordonii*, *S. mitis*, and *S. salivarius*) were conducted. As shown in Figure 4a, the MICs of LH12 against *S. mutans*, *A. viscosus*, *A. naeslundii*, *L. casei*, and *L. fermentum* were from 8.00 μg/mL to 16.00 μg/mL, and the MBCs were from 16.00 μg/mL to 32.00 μg/mL. The MIC of LH12 against *S. salivarius* was 48.00 μg/mL and the MBC was 64.00 μg/mL. The MICs and MBCs of LH12 against *S. sanguinis*, *S. gordonii* and *S. mitis* were no less than 128.00 μg/mL. The tested cariogenic bacteria were much more susceptible to LH12 than the commensal bacteria. As shown in Figure 4b, LH12 displayed greater growth inhibitory effects on cariogenic bacteria. LH12 at 8 μg/mL delayed the growth of *S. mutans*, *A. naeslundii* and *L. casei*, and inhibited the growth of *A. viscosus* and *L. fermentum*. LH12 at 16 μg/mL totally inhibited the cariogenic species. On the contrary, 16 μg/mL LH12 did not affect the growth of *S. sanguinis*, *S. gordonii*, *S. mitis* and *S. salivarius*, and 32 μg/mL LH12 slightly influenced the growth patterns of the commensal streptococci. The above results demonstrated the selective inhibitory effects of LH12 on the cariogenic bacteria.

### Mechanisms of the acid-activated property of LH12

The most commonly cited models for the antimicrobial activity of cationic amphipathic α-helical antimicrobial peptides involve that the mixed cationic and hydrophobic composition of peptides make them suited for interacting and perturbing bacterial cytomembranes, and the accumulation of peptides with specific structures permeabilize and kill bacteria [29]. Therefore, the mechanisms of the acid-activation were demonstrated upon the electrostatic interaction, shift of the secondary structure, perturbance of cytomembranes, and permeabilizing activity.

**Figure 5.**
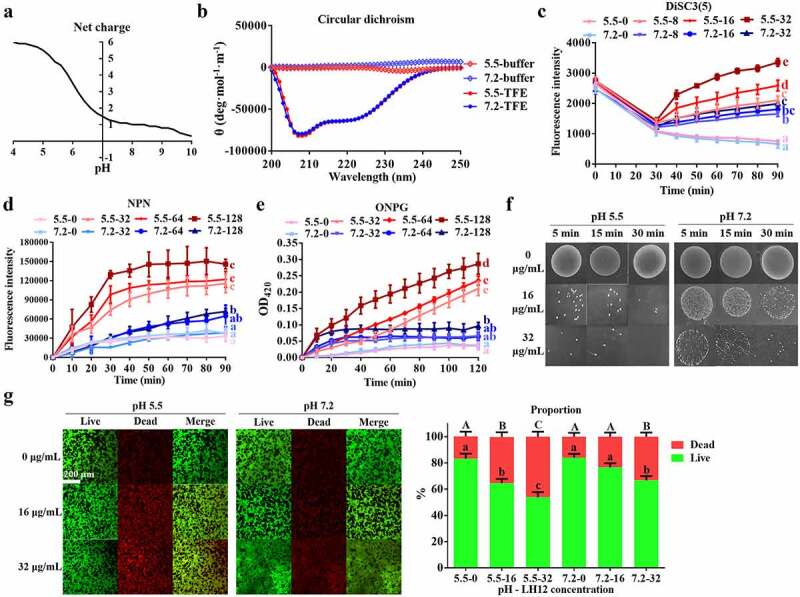
Mechanisms of the pH-responsive property of LH12. (a) net charge/pH curve of LH12. (b) circular dichroism spectra of LH12 at pH 5.5 and pH 7.2. (c) membrane depolarization activity of LH12 were tested using DiSC3(5) at pH 5.5 and pH 7.2. Data are represented as mean ± standard deviation. Different superscript letters at the endpoints indicate significant differences (*P* <0.05). (d) outer membrane permeabilization was measured using NPN at pH 5.5 and pH 7.2. Data are represented as mean ± standard deviation. Different superscript letters at the endpoints indicate significant differences (*P* <0.05). (e) inner membrane permeabilization was measured using ONPG at pH 5.5 and pH 7.2. Data are represented as mean ± standard deviation. Different superscript letters at the endpoints indicate significant differences (*P* <0.05). (f) spot assay of *S. mutans* at pH 5.5 and pH 7.2. (g) representative images of Live/Dead staining and biomass percentages of live and dead bacteria of the preformed *S. mutans* biofilms treated with LH12 at pH 5.5 and pH 7.2. Data are represented as mean ± standard deviation. Different superscript letters indicate significant differences (*P* <0.05).

LH12 displayed an increase of net charge with lowering of pH values (Figure 5a), which was because of the protonation of histidine in acidic condition [30]. The increased cationicity would help LH12 attach to and accumulate on the anionic microbial cytomembranes through electrostatic interactions. The secondary structure of peptides may change from a random coil at neutral pH to a helix at acidic pH and the structure shift can enhance the antibacterial effect [31]. The CD spectra verified the α-helix of LH12 with typical double minima at 208 nm and 222 nm in the TFE solution simulating the lipid membrane environment (Figure 5b). Figure 5b also shows that the CD spectra of LH12 at pH 5.5 and pH 7.2 were the same in both phosphate/citrate buffer and TFE solution, suggesting that the structure change might not contribute to the acid-activated activity. The fluorescence intensity of DiSC3(5) will increase when the membranes are depolarized and perturbed [31]. LH12 at pH 5.5 had a more pronounced effect on depolarizing bacterial cytomembranes than at pH 7.2 (Figure 5c), indicating the intensified perturbing ability of LH12 in the acidic condition. Intact outer membranes can block NPN and quench its fluorescence. However, LH12 at pH 5.5 induced a rapider and higher increase of fluorescence intensity (Figure 5d), indicating that the acidic condition promoted LH12 to permeabilize the outer membrane. β-galactosidase inside bacteria interact with ONPG to produce *o*-Nitrophenyl when an internal and external permeation occurs. The OD_420_ values at pH 5.5 increased more significantly than that at pH 7.2 (Figure 5e), indicating that the acidic condition enhanced the capacity of LH12 to cause membrane disruption. As shown in Figure 5f, much less bacterial colonies were observed after the treatment of LH12 at pH 5.5 than at pH 7.2. The stains (SYTO-9/PI) assessing membrane integrity were used to distinguish between membrane-intact and membrane-injured cells. Bacteria with intact cell membranes (live bacteria) were stained green (SYTO-9) while bacteria with damaged membranes (dead bacteria) were stained red (PI). An increase of red fluorescence was observed after the treatment of LH12 at pH 5.5, while the biofilms in the groups at pH 7.2 were almost green (Figure 5g). The relative quantification of the Live/Dead ration also demonstrated that LH12 at pH 5.5 killed more *S. mutans* embedded in the biofilm. The results of Figures 5f and 5g demonstrated the acid-activated antibacterial and antibiofilm effects.

The mechanisms of the acid-activated property of LH12 are summed up as follows: The acidic pH protonates the histidine residues of LH12; the increased cationicity enhances its attachment to bacterial cytomembranes via electrostatic attraction; the attached LH12 perturbs bacterial membranes more effectively and results in the increased permeation of bacteria; and LH12 subsequently exhibits a more potent killing effect against the bacteria and biofilms in response to the acidified microenvironment.

### Effects and mechanisms of LH12 on suppressing the virulence of S. mutans

**Figure 6.**
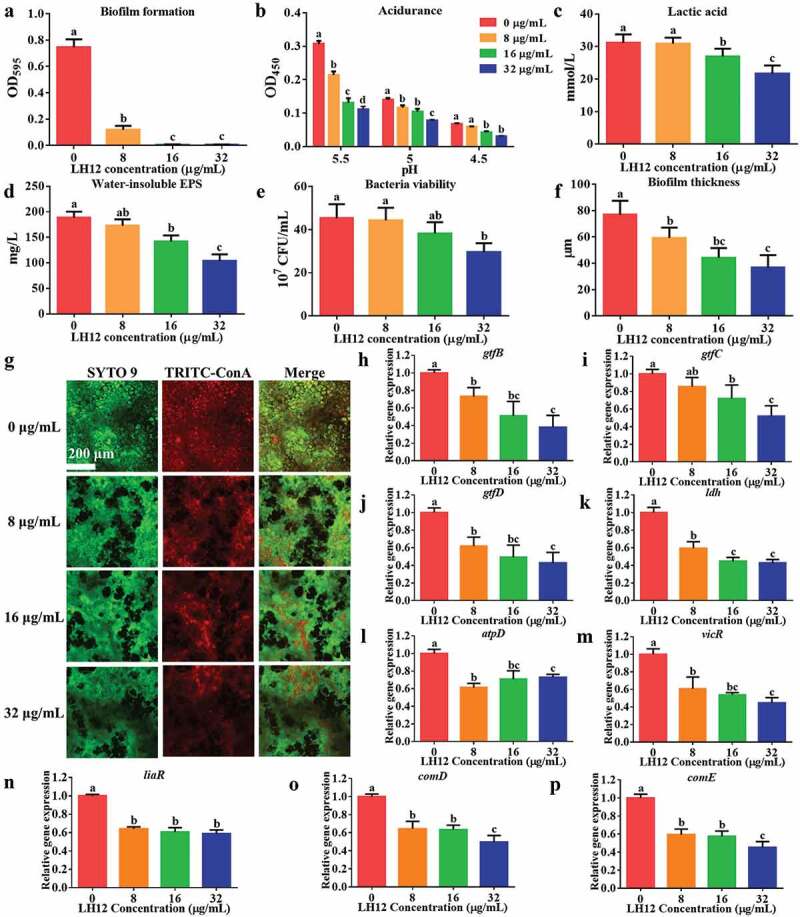
Measurement of biofilm formation (a), acidurance (b), lactic acid production (c), water-insoluble EPS synthesis (d), bacteria viability (e) and biofilm thickness (f) was conducted to evaluate the effects of LH12 on the cariogenic virulence of *S. mutans*. Data are represented as mean ± standard deviation. Different superscript letters indicate significant differences (*P* <0.05). (g) Representative CLSM images of the preformed *S. mutans* biofilms exposed to the short-term treatment of LH12. Bacteria were stained with SYTO-9 and EPS were stained with TRITC-ConA. The expression of the virulence genes, such as *gtfB* (h), *gtfC* (i), *gtfD* (j), *ldh* (k) and *atpD* (l), and the genes of TCSTS, such as *vicR* (m), *liaR* (n), *comD* (o) and *comE* (p) were relatively quantified using 2^−ΔΔCt^ method. Data are represented as mean ± standard deviation. Different superscript letters indicate significant differences (*P* <0.05).

*S.mutans* is a classic model with typical cariogenic virulence factors, such as acidogenicity, acidurance and biofilm formation, to evaluate the anticaries potential [6]. Figure 6a shows that 8 μg/mL LH12 could reduce 84.04% of biofilm mass, and 16 μg/mL and 32 μg/mL LH12 totally inhibited the biofilm formation. Acidurance is a critical virulence factor that helps *S. mutans* survive and metabolize in the acidified microenvironment, while Figure 6b shows that the vitality of *S. mutans* in low pH condition (pH 5.5, pH 5, pH 4.5) decreased significantly after 5-min short-term treatment of LH12. The biochemical properties of the preformed biofilm provide bacteria with the protection against antimicrobials, posing significant challenges for the development of effective antimicrobial therapeutics to control dental caries [7]; however, the short-term treatment of LH12 could suppress the cariogenic properties of the preformed biofilms. Figures 6c and 6d show that 16 μg/mL and 32 μg/mL LH12 could decrease the lactic acid production and water-insoluble EPS synthesis of the preformed biofilms. Figure 6e shows that 32 μg/mL LH12 reduced the viable *S. mutans* in the preformed biofilms. Figure 6f shows that LH12 at no less than 8 μg/mL inhibited biofilm accumulation and 32 μg/mL LH12 reduced the biofilm thickness to a half of that in the control group. The CLSM images demonstrated that the viable bacteria stained by SYTO-9 and EPS stained by TRITC-ConA decreased with the increase of LH12 (Figure 6g). Besides, as shown in Figure 6g, the preformed biofilms treated with LH12 were loose and porous, whereas that of the control group showed a high-density of bacteria embedded with massive extracellular matrix. In conclusion, LH12 could inhibit various cariogenic virulence factors of *S. mutans*.

Interestingly, although 8 μg/mL was a sub-MIC at which LH12 could not kill *S. mutans* theoretically, 8 μg/mL LH12 still suppressed acidurance, biofilm thickness and biofilm integrity, which suggested other mechanisms of the inhibitory effects on the cariogenic virulence besides of the direct killing of *S. mutans*. Antimicrobial peptides can also act on DNA, RNA and proteins to disturb the vitality and metabolism [32]. Here, we further explore the inhibitory mechanisms at transcriptional level.

Glucosyltransferases are crucial to bacterial adhesion and biofilm formation [33]. LH12 reduced the expression of *gtfBCD* (Figure 6h-j) and accordingly inhibited the formation and integrity of biofilms. Lactate dehydrogenase is an important enzyme of glycolysis [26]. LH12 reduced the expression of *ldh* (Figure 6k) and accordingly inhibited the acidogenicity of *S. mutans*. The expression of *atpD*, a gene encoding alpha-subunit of F-type ATPase that helps maintain cytoplasmic pH, was also reduced (Figure 6l), which could result in the inhibition of acidurance. VicRK, LiaSR and ComDE systems are parts of the two-component signal transduction systems (TCSTS), which are key factors involved in sensing and responding to environmental stresses and dictate survival in hostile niches, which are also related to the expression of multiple virulence factors [34]. LH12 could significantly downregulate the gene expression of TCSTS, such as *vicR*, *liaR* and *comDE* (Figure 6m-p).

### Effects and mechanisms of LH12 on regulating the dual-species biofilms

**Figure 7.**
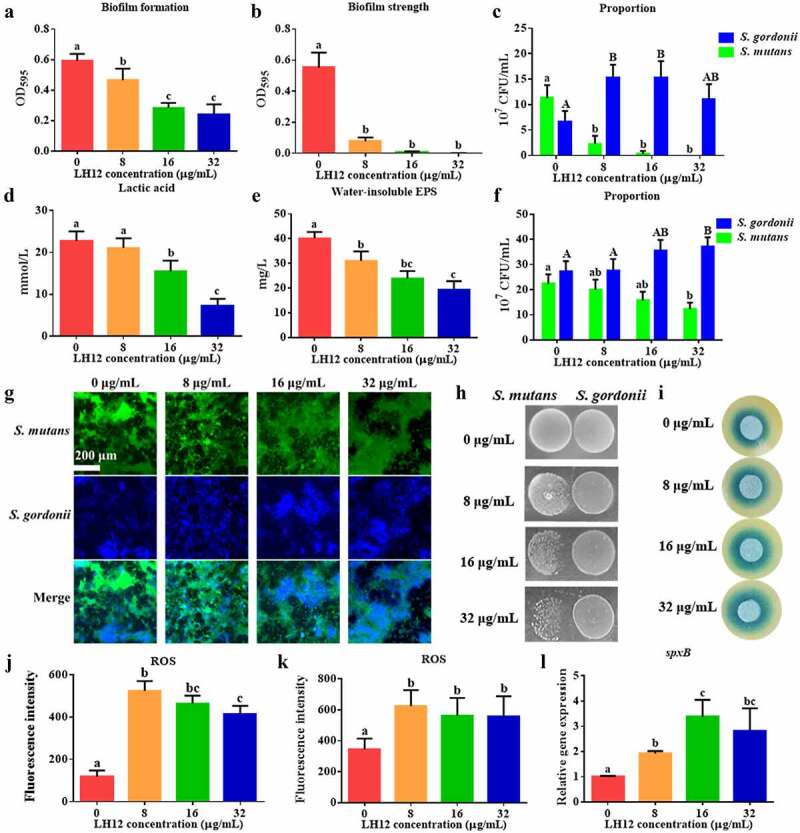
Biofilm formation (a), biofilm strength (b) and bacterial composition (c) of the 24-hour-old dual-species biofilms, and lactic acid production (d), water-insoluble EPS synthesis (e) and bacterial composition (f) of the preformed dual-species biofilms exposed to the short-term treatment of LH12 were measured to evaluate the regulating effects of LH12 on the biofilms containing both cariogenic bacteria and commensal bacteria. Data are represented as mean ± standard deviation. Different superscript letters indicate significant differences (*P* <0.05). (g) representative FISH images of the preformed dual-species biofilms exposed to the short-term treatment of LH12. (h) competition between *S. mutans* and *S. gordonii* on half-strength BHI plates with the treatment of LH12. (i) H_2_O_2_ production of *S. gordonii* with the treatment of LH12 were observed using Prussian blue plates on which blue halos indicate the quantity of H_2_O_2_. ROS level of *S. gordonii* (j) and the dual-species biofilms (k) were measured using DCFH-DA. Data are represented as mean ± standard deviation. Different superscript letters indicate significant differences (*P* <0.05). (l) the expression of *spxB*, which is involved in H_2_O_2_ production, was relatively quantified using 2^−ΔΔCt^ method. Data are represented as mean ± standard deviation. Different superscript letters indicate significant differences (*P* <0.05).

Considering that LH12 could target cariogenic bacteria without perturbing commensal bacteria, a dual-species model containing *S. gordonii* and *S. mutans* was used to explore if the pH-responsive antimicrobial peptide could regulate the microecology [3,35]. As shown in Figures 7a and 7b LH12 could inhibit the formation of the dual-species biofilm and reduce its strength to make it easier to remove. The proportion of *S. gordonii* in the 24-hour-old dual-species biofilm significantly increased in the 8 mg/L LH12 group and the biofilms were composed mainly of *S. gordonii* under the treatment of 16 mg/L and 32 mg/L LH12 (Figure 7c), suggesting that LH12 could selectively inhibit the cariogenic bacteria without affecting the colonization of the commensal bacteria in the mixed-species microbial community. The short-term treatment of LH12 reduced the lactic acid production (Figure 7d) and water-insoluble EPS synthesis (Figure 7e) of the dual-species biofilm. According to the CFU counting (Figure 7f) and FISH (Figure 7g), the microbial composition of the preformed dual-species biofilms was regulated to a condition with an increased proportion of *S. gordonii* and a reduced proportion of *S. mutans*, which demonstrated the ecological regulation effect of LH12

Commensal streptococci could antagonize against *S. mutans* to help maintain the ecological equilibrium of dental plaque [3]. The growth of *S. mutans* was inhibited by *S. gordonii* with the treatment of LH12 (Figure 7h), suggesting that LH12 helped promote the competitiveness of *S. gordonii*. The enlarged blue halos around *S. gordonii* with the treatment of LH12 demonstrated the increased production of H_2_O_2_ (Figure 7i). Intrinsic H_2_O_2_ is a crucial regulatory molecule in the establishment of dental plaque biofilms, which favors commensal growth and helps maintain dental health [4]. Figures 7j and 7k show that LH12 increased the generation of ROS, which could facilitate the suppression of *S. mutans*. LH12 also upregulated the expression of *spxB*, a gene encodes pyruvate oxidase to catalyze the synthesis of H_2_O_2_ (Figure 7l) [36].

## Discussion

The design of LH12 was inspired by the formula (XXYY)_n_ for cationic amphiphilic α-helical antimicrobial peptides, in which X refers to hydrophobic residues, Y refers to hydrophilic residues and n refers to the repeats [13,37]. Leu was chosen as the hydrophobic residue for it often occurs in synthetic antimicrobial peptides, and His was chosen as the hydrophilic residue because of its pH-sensitivity [11]. Gly was present at the first position to act as an efficient N-capping, and C-terminus was amidated to increase antimicrobial activity [13,38]. Arginine and lysine are often chosen as the alkaline residues in synthetic antimicrobial peptides because these positively charged residues help target and kill bacteria [39]. Histidine is largely unprotonated and uncharged at physiological pH and the replacement of lysine and arginine by histidine could abolish the antimicrobial capacity of certain peptides [11]. However, the electron lone pair in the unsaturated nitrogen of histidine endows histidine-rich peptides with the capacity to be cationic at acidic pH and thus the lowering of pH restores the antibacterial activity [30]. The histidine-rich sequence endowed LH12 with the pH-responsive property as expected.

Several precision-guided antimicrobial peptides were successfully designed to specifically target *S. mutans* in oral biofilms [40–42]. Although *S. mutans* is still recognized as the primary etiological agent of dental caries, it is not the only determinant. In many cases, the proportion of *S. mutans* is not strictly correlated with dental caries [43,44]. It is not one particular bacterium, but multiple microorganisms with similar physiological properties, such as acid production and acid tolerance, that contribute collectively to the development of dental caries. Therefore, a pH-responsive antimicrobial peptide that can target multiple cariogenic pathogens may be more aligned with the requirements of the ecological approaches to caries prevention. The results of Figure 3 verify that LH12 could selectively inhibit multiple cariogenic bacteria and suggested its potential as an ecological approach to dental caries. Figures 5f and 5g imply the smartness of LH12 that LH12 would not disturb the niches within the physiological pH range and would affect the acidified niches with a tendency to develop into the cariogenic microenvironment. The acid-activated pH-responsive mechanism endowed LH12 with not only the target of cariogenic pathogens but also the target of the acidified niches with high risk of dental caries [9,45]. To further explain the different antibacterial effects of LH12 on cariogenic bacteria and commensal bacteria, a cell surface hydrophobicity assay was conducted. The reduction of cell surface hydrophobicity indicates the modification and instability of the cell membrane [46,47]. As shown in Figure S1, the hydrophobicity rate of *S. mutans* was significantly lower than *S. gordonii*, suggesting the stronger membrane disturbing potential of LH12 on *S. mutans* and partially explaining the preferentially inhibitory effects on the cariogenic bacteria. The selective inhibitory effects of LH12 may owe to its pH-responsive property and its stronger interactions with cariogenic bacteria, which remains to be further explored.

**Figure 8.**
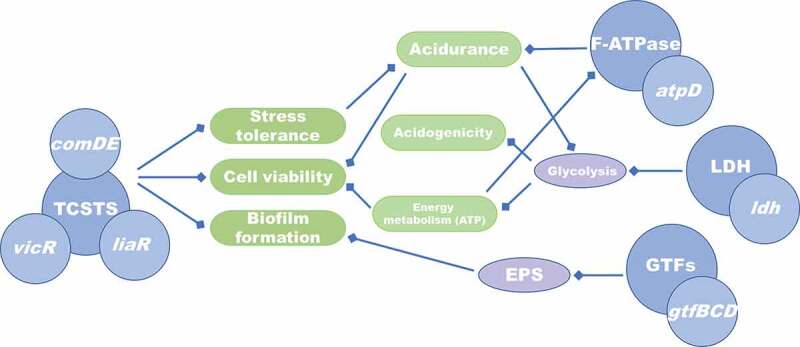
Inhibitory effects of LH12 on the regulatory network of multiple genes.

Interactions among the virulence factors might explain the broad inhibitory effects of LH12 on various cariogenic properties. For example, the downregulation of *atpD* could lead to cytoplasmic acidification and inhibit the normal process of glycolysis, which further suppressed acid production and disturbed energy metabolism to affect the cell viability. The downregulation of *ldh* could inhibit glycolysis and reduce the generation of ATP, which in turn decreased the activity of F-type ATPase and suppressed the acidurance. VicRK system can regulate the sucrose-dependent biofilm formation and acid production; the deficiency in ComDE system can result in the lack of architectural integrity of biofilms; and VicRK, ComDE and LiaSR systems contribute to the acid tolerance [34,48]. LH12 could downregulate the expression of *vicR*, *liaR* and *comDE* to inhibit the action of TCSTS to suppress the virulence of *S. mutans*. LH12 suppressed the cariogenic virulence factors of *S. mutans* not only by inhibiting the expression of particular genes but also by influencing the regulatory networks of multiple genes (Figure 8).

Commensal streptococci, such as *S. sanguinis*, *S. gordonii*, *S. mitis* and *S. salivarius*, can produce alkali to neutralize acid and generate H_2_O_2_ and bacteriocins to inhibit the persistence of cariogenic bacteria, which plays an important role in the acid-base equilibrium of tooth surfaces and microecological homeostasis of oral microflora [49]. The selective inhibition of LH12 on the cariogenic pathogens without perturbing commensal bacteria suggested its potential microecological regulation effect. LH12 could weaken the resistance of *S. mutans* to commensal bacteria. For example, ComDE, LiaSR and VicRK systems are involved in tolerating various stressors, modulating mutacins production to compete with commensal streptococci and maintaining cell viability [50–52]. The downregulation of *vicR*, *liaR* and *comDE* could inhibit the antagonism of *S. mutans* against *S. gordonii* and its resistance to H_2_O_2_. Besides of the suppression on *S. mutans*, LH12 could also promote *S. gordonii* to produce H_2_O_2_ and generate ROS to enhance its competitiveness. The above results demonstrated the dual mode of the ecological regulation effect of LH12 that LH12 suppressed the activity of *S. mutans* while promoting the ecological advantages of *S. gordonii*. Antimicrobial agents usually prevent dental caries by inhibiting cariogenic bacteria and subsequently improve the advantages of commensal bacteria relatively; however, LH12 itself could directly promote the competitiveness of commensal bacteria. It seems instructive that the effects on both cariogenic bacteria and commensal bacteria should be explored when evaluating an anticaries agent and that promoting the ecological advantages of commensal bacteria is an alternative strategy for caries prevention. Although dual-species models were established to explore the regulation effect, there still remain limitations in the current study that could be addressed by future work. Considering the complex microbial community composition of the dental plaque biofilms and complex physiochemical properties of the oral environment, a more complex multispecies biofilm model *in vitro* and rat caries model *in vivo* should be utilized to further evaluate the ecological regulation effects of LH12.

**Figure 9.**
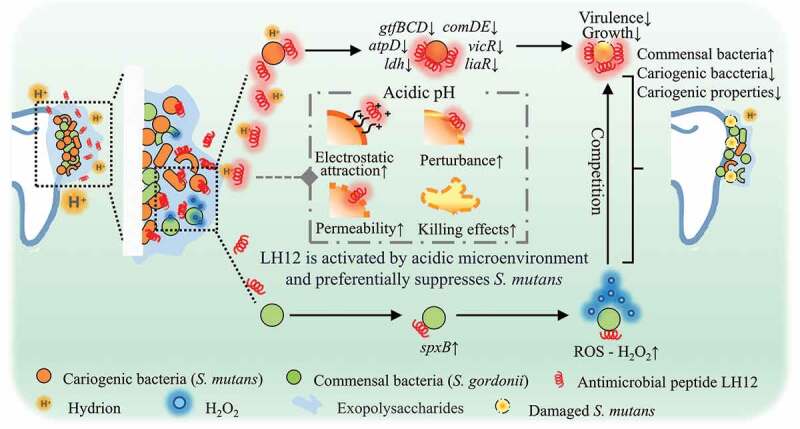
Schematic illustration of the dual-functional mechanism of LH12 on suppressing the cariogenic properties and regulating the bacterial composition of the biofilms. LH12 could target the cariogenic pathogens via being activated by the acidic microenvironment and inhibit their cariogenic virulence and expression of the related genes. In addition, LH12 could also improve the ecological competitiveness of the commensal bacteria by promoting the production of H_2_O_2_.

In conclusion, a novel bioresponsive ecological approach to dental caries prevention was introduced. The pH-responsive antimicrobial peptide LH12 was protonated in the acidified cariogenic microenvironment, showed higher cationicity and stronger interactions with bacterial membranes, and subsequently exhibited enhanced antibacterial and antibiofilm effects in the acidic condition. The acid-activated mechanism endowed LH12 with the capacity to target the cariogenic pathogens and the acidified niches without perturbing the commensal bacteria. LH12 could inhibit various cariogenic virulence factors of *S. mutans*, such as biofilm formation, acidurance, acid production and polysaccharides synthesis. The virulence genes, such as *gtfBCD*, *atpD* and *ldh*, were downregulated. Simultaneously, the gene expression of TCSTS that are related with stress tolerance, cell viability and regulation of cariogenic virulence, such as *vicR*, *liaR* and *comDE*, was also decreased. Furthermore, LH12 promoted *S. gordonii* to produce H_2_O_2_, increased the intrinsic ROS, enhanced the antagonism against *S. mutans*, improved the ecological advantages of the commensal bacteria and regulated the bacterial composition of the dual-species biofilms. In brief, LH12 regulated the microbial communities and suppressed the cariogenic properties of the biofilms through a dual-functional mechanism that LH12 selectively inhibit cariogenic bacteria via being activated by the acidic microenvironment and improved the ecological competitiveness of commensal bacteria (Figure 9). The results of this study may lead to a novel bioresponsive ecological approach for caries prevention that specifically targets pathogens in a mixed species microbial community without disturbing commensals and regulates the biofilm to a healthier condition with lower cariogenic properties.

## Supplementary Material

Supplemental Material
