# Supplementary material for: Targeting cariogenic pathogens and promoting competitiveness of commensal bacteria with a novel pH-responsive antimicrobial peptide
Source: J Oral Microbiol. 2022 Dec 20;15(1):2159375. doi: 10.1080/20002297.2022.2159375 (PMC9788686; doi:10.1080/20002297.2022.2159375)
Supplement: Supplemental Material [file ZJOM_A_2159375_SM3474.docx]

1. Cell surface hydrophobicity assay

*S. mutans* and *S. gordonii* (10^9^ CFU/mL) were washed with PBS and resuspended in phosphate urea magnesium sulphate buffer (100 mmol/L K_2_HPO_4_, 50 mmol/L KH_2_PO_4_, 30 mmol/L urea, and 0.8 mmol/L MgSO_4_·7H_2_O). The suspensions were incubated with LH12 at 37 ℃ for 10 min, and the initial absorbance was measured using a Synergy H1 multi-mode microplate reader at 550 nm. Aliquots of bacterial suspensions were mixed with hexadecane. The mixture was shaken for 5 min and allowed to stand for 15 min to complete the phase separation. The final absorbance of the aqueous phase of the mixture was measured at 550 nm. The surface hydrophobicity of the bacteria was expressed as the percentage reduction of the OD_550_ values of the aqueous phase.


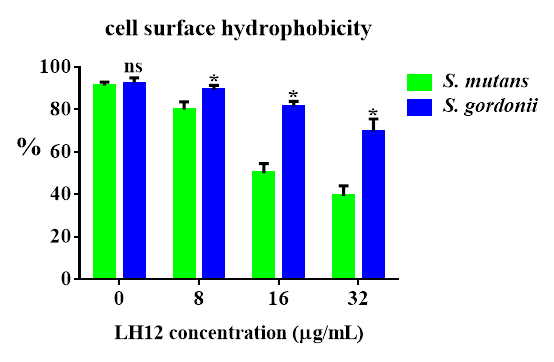


**Figure S1.** Cell surface hydrophobicity assay. Cell surface hydrophobicity (%) = (OD_550-initial_ – OD_550-final_)/OD_550-initial_ × 100%. The cell surface hydrophobicity of *S. mutans* and *S. gordonii* within each concentration group was analyzed individually using the Holm-Sidak t-test. Data are represented as mean ± standard deviation. *: *P* < 0.05, ‘ns’: no significance (*P* > 0.05).

1. Quantitative real-time PCR

Mid-logarithmic phase cultures of *S. mutans* and *S. gordonii* were centrifugated (8000 rpm, 5 min). The bacteria were resuspended in fresh BHI broth to an OD_600_ of 0.2 and incubated with or without LH12 for 30 min at 37 ℃ anaerobically. RNA was extracted and purified using an RNA-Quick Purification Kit (Yishan, China). The concentration and quality of the RNA samples were determined by a Nanodrop 2000 spectrophotometer (Thermo Fisher Scientific, USA). Reverse transcription was then performed using a PrimeScript^TM^ RT reagent Kit with gDNA Eraser (TaKaRa, Japan). The mixture [4 μL PrimeScript^TM^ RT Master Mix, ‘X’ μL RNA (X = 1000 ng / RNA concentration) and ‘16−X’ μL nuclease-free water] was incubated using an Applied Biosystems^®^ Veriti^®^ 96-Well Fast Thermal Cycler (Thermo Fisher Scientific, USA) following the procedure (37 ℃ 15 min → 85 ℃ 5 s → 4 ℃). The concentration and quality of the cDNA samples were determined by a Nanodrop 2000 spectrophotometer. The cDNA samples were diluted to a concentration of 100 ng/μL with nuclease-free water. Tested genes and specific primers were listed in the table below. The specificity was checked by BLAST. The primers were synthesized TsingKe BioTech (China). Quantitative real-time PCR was performed with Hieff® qPCR SYBR^®^ Green Master Mix (Yeasen, China). The mixture (6 μL nuclease-free water, 2 μL cDNA, 10 μL SYBR^®^ Green Master Mix, 1 μL forward primer and 1 μL reverse primer) was incubated using a LightCycler® 480-II system (Roche, Switzerland). PCR procedures were set as follows: holding stage (95 °C, 30 s); cycling stage for 40 cycles (denaturation: 95 °C, 10 s; annealing: 60 °C, 20 s; extension: 72 °C, 20 s); melt curve stage (95 °C, 5 s; 65 °C, 1 min; 97 °C, 5 s); cool down stage (40 °C, 10 s). The relative gene expression fold changes were calculated with 2^−ΔΔCt^ method with values normalized to the reference gene 16S rRNA. The experiment was performed in triplicate and repeated three times.

Primers used in quantitative real-time PCR.

| **Primers** |  | **Sequences (5’-3’)** | **PCR product size** | **GC%** | **Tm** **℃** | **Reference** |
| --- | --- | --- | --- | --- | --- | --- |
| *S. m* 16S rRNA | F | AGCGTTGTCCGGATTTATTG | 157 | 45 | 56.51 | [1] |
|  | R | CTACGCATTTCACCGCTACA |  | 50 | 58.36 |  |
| *ldh* | F | AAAAACCAGGCGAAACTCGC | 255 | 50 | 59.97 | [1] |
|  | R | CTGAACGCGCATCAACATCA |  | 50 | 59.56 |  |
| *atpD* | F | TGTTGATGGTCTGGGTGAAA | 176 | 45 | 56.96 | [1] |
|  | R | TTTGACGGTCTCCGATAACC |  | 50 | 57.34 |  |
| *gtfB* | F | CACTATCGGCGGTTACGAAT | 194 | 50 | 57.87 | [1] |
|  | R | CAATTTGGAGCAAGTCAGCA |  | 45 | 56.91 |  |
| *gtfC* | F | GATGCTGCAAACTTCGAACA | 164 | 45 | 57.31 | [1] |
|  | R | TATTGACGCTGCGTTTCTTG |  | 45 | 57.13 |  |
| *gtfD* | F | TTGACGGTGTTCGTGTTGAT | 219 | 45 | 58.06 | [1] |
|  | R | AAAGCGATAGGCGCAGTTTA |  | 45 | 57.98 |  |
| *vicR* | F | CGTGTAAAAGCGCATCTTCG | 220 | 50 | 58.21 | [1] |
|  | R | AATGTTCACGCGTCATCACC |  | 50 | 59.20 |  |
| *liaR* | F | CATGAAGATTTAACAGCGCG | 202 | 45 | 55.85 | [1] |
|  | R | CGTCCTGTGGCACTAAATGA |  | 50 | 57.91 |  |
| *comD* | F | TTCCTGCAAACTCGATCATATAGG | 113 | 41.67 | 58.46 | [1] |
|  | R | TGCCAGTTCTGACTTGTTTAGGC |  | 47.83 | 61.30 |  |
| *comE* | F | TTCCTCTGATTGACCATTCTTCTG | 147 | 41.67 | 58.51 | [1] |
|  | R | GAGTTTATGCCCCTCACTTTTCAG |  | 45.83 | 59.84 |  |
| *S. g* 16S rRNA | F | AAGCAACGCGAAGAACCTTA | 194 | 45 | 58.13 | [1] |
|  | R | GTCTCGCTAGAGTGCCCAAC |  | 60 | 60.46 |  |
| *spxB* | F | GGATGCTTTGGCTGAAGAC | 129 | 52.63 | 56.59 | [2] |
|  | R | GGACCACCTGAACCTACTG |  | 57.89 | 56.76 |  |

1. Wang Y, Wang X, Jiang W, et al. Antimicrobial peptide GH12 suppresses cariogenic virulence factors of Streptococcus mutans. J Oral Microbiol. 2018;10(1):1442089. doi: 10.1080/20002297.2018.1442089. PubMed PMID: 29503706; PubMed Central PMCID: PMCPMC5827641.

2. Jiang W, Wang Y, Luo J, et al. Effects of Antimicrobial Peptide GH12 on the Cariogenic Properties and Composition of a Cariogenic Multispecies Biofilm. Appl Environ Microbiol. 2018 Dec 15;84(24). doi: 10.1128/AEM.01423-18. PubMed PMID: 30341079; PubMed Central PMCID: PMCPMC6275336.
